# Cost minimization analysis of a hexavalent vaccine in Argentina

**DOI:** 10.1186/s12913-023-10038-0

**Published:** 2023-10-06

**Authors:** Ignacio Olivera, Carlos Grau Pérez, Luis Lazarov, Eduardo Lopez, Cristian Oddo, Hugo Dibarboure

**Affiliations:** 1 Centro de Investigaciones Económicas, CINVE, Salud, Montevideo, Uruguay; 2 Facultad de Medicina, Universidad del Salvador, Buenos Aires, Argentina; 3 Sanofi Vaccines, Buenos Aires, Argentina; 4 Sanofi Vaccines, Montevideo, Uruguay; 5 Cuyo 3512, Martinez, Buenos Aires, B1640GIY Argentina

**Keywords:** Vaccination, Cost-minimization, Pediatric, Health outcomes, Pertussis, Diphtheria, Tetanus, Polio, Hepatitis B, *Haemophilus influenza* type B

## Abstract

**Background:**

Argentina currently uses a pentavalent vaccine containing diphtheria, tetanus, pertussis (whole cell), *Haemophilus influenza* type b and hepatitis B antigens, administered concomitantly with the inactivated polio vaccine (IPV) (DTwP-Hib-HB plus IPV) in its childhood vaccination schedule. However, hexavalent vaccines containing acellular pertussis antigens (DTaP-Hib-HB-IPV) and providing protection against the same diseases are also licensed, but are only available with a private prescription or for high-risk pre-term infants in the public health program. We analyzed the cost of switching from the current schedule to the alternative schedule with the hexavalent vaccine in Argentina, assuming similar levels of effectiveness.

**Methods:**

The study population was infants ≤ 1 year of age born in Argentina from 2015 to 2019. The analysis considered adverse events, programmatic, logistic, and vaccine costs of both schemes from the societal perspective. The societal costs were disaggregated to summarize costs incurred in the public sector and with vaccination pre-term infants in the public sector. Costs were expressed in 2021 US Dollars (US$).

**Results:**

Although the cost of vaccines with the alternative scheme would be US$39.8 million (M) more than with the current scheme, these additional costs are in large part offset by fewer adverse event-associated costs and lower programmatic costs such that the overall cost of the alternative scheme would only be an additional US$3.6 M from the societal perspective. The additional cost associated with switching to the alternative scheme in the public sector and with the vaccination of pre-term infants in the public sector would be US$2.1 M and US$84,023, respectively.

**Conclusions:**

The switch to an alternative scheme with the hexavalent vaccine in Argentina would result in marginally higher vaccine costs, which are mostly offset by the lower costs associated with improved logistics, fewer separate vaccines, and a reduction in adverse events.

**Supplementary Information:**

The online version contains supplementary material available at 10.1186/s12913-023-10038-0.

## Background

Immunization programs have been fundamental in reducing morbidity and mortality associated with many communicable diseases worldwide. For example, the use of polio vaccines since their introduction in the 1950’s has brought the near global eradication of the disease [1]. Moreover, increased access to immunization, especially in developing countries, as well as the introduction of new vaccines overtime, has helped lower the mortality rate in those under-5 years old from 17 million in 1970 to 5 million in 2020 [2, 3]. Combination vaccines have played a significant role in improving immunization coverage by allowing individuals to be vaccinated against multiple communicable diseases simultaneously, whilst simplifying programmatic and logistic requirements [4–6].

The Argentinean Ministry of Health created the Immunization National Commission as a scientific advisory group in 2000 to prioritize vaccinations [7]. The intention was to inform and strengthen the actions of the National Immunization Program in making evidence-based decisions regarding vaccines and immunizations when formulating recommendations in their goal to control and, whenever possible, eliminate/eradicate vaccine-preventable diseases. In 2009, a pentavalent vaccine containing diphtheria, tetanus, pertussis (whole-cell), *Haemophilus influenza* type b and hepatitis B (DTwP-Hib-HB) antigens was introduced in Argentina [8]. Currently, children receive the pentavalent vaccine at age 2, 4 and 6 months with a booster at age 15–18 months [9]. The inactivated polio vaccine (IPV) is also administered concomitantly with the pentavalent vaccine at 2, 4 and 6 months, with an IPV booster at 5 years [10]. However, there are also two hexavalent vaccines (containing acellular pertussis antigens) protecting against the six mentioned diseases currently licensed in Argentina: Hexaxim^®^ (Sanofi) and Infanrix-Hexa^®^ (GSK). Both hexavalent vaccines are only available with either a private prescription, or through the Pan American Health Organization (PAHO) for high-risk, pre-term infants in the public health program.

Despite the well-established Argentinean childhood vaccination program, coverage with the third dose of DTwP-Hib-HB in the country according to WHO/UNICEF data decreased from 83% prior to the COVID-19 pandemic to 76% (or to 81% according to official/administrative reported coverage) in 2021, with reductions in coverage with most other vaccines also observed, and with similar trends noted in other Latin American countries [11]. Globally, an estimated 25 million children were unvaccinated or under-vaccinated in 2021 [12]. Many factors are thought to have contributed to the decline in non-SARS-CoV-2 vaccinations globally, including increased vaccine misinformation, an increased number of children living in troubled settings, and COVID-19 related issues including service and supply chain disruptions, resource diversion and containment measures that limited access and availability of vaccination services [12].

Advancements in healthcare innovations increasingly necessitates that health outcome evaluations are accompanied by economic assessments to help inform decision-making about their financing. Switching to the hexavalent vaccine would be expected to simplify logistics and associated delivery infrastructure, reduce the number of injections and side effects, as well as minimize administration error which may result in better acceptability, convenience and compliance, and ultimately improved vaccination coverage [13]. Previous studies assessing the potential economic impact of introducing the hexavalent vaccine in the national childhood vaccination programs of Peru, Colombia, and Chile suggest that it would lead to additional acquisition costs, which are partial mitigated by improved logistics, and reduced incidence of adverse events [14–16]. Here, we assessed the differences in cost from switching from the current childhood primary and booster vaccination schedule with the pentavalent vaccine plus IPV (DTwP-Hib-HB plus IPV) to an alternative series with the hexavalent vaccine (DTaP-Hib-HB-IPV) in Argentina, assuming similar levels of effectiveness across the six biologicals of the vaccine.

### Objectives

The objective of this study was to estimate the cost difference between the current childhood vaccination scheme in Argentina consisting of a pentavalent vaccine plus a polio vaccine (DTwP-Hib-HB plus IPV) (see below), compared to an alternative scheme with the hexavalent vaccine (DTaP-Hib-HB-IPV) from the societal perspective. The societal perspective considers all costs relevant to society incurred such as costs related to healthcare (out/in-patient services in the public, social security, and private sectors), interventions, logistics, as well as costs associated with lost resources (e.g. absenteeism), patient-related travel, premature death, and informal care. In addition, we disaggregated the societal costs to summarize those incurred in the public sector and with the vaccination of pre-term infants in the public sector.

## Methods

### Population

The study population for both schemes was composed of infants up to 1 year of age born in Argentina, taking the average annual cohort of newborns from 2015 to 2019 reported by the Argentinean Ministry of Health and publicly available without restrictions [17–21]. The five-year annual average was 44.0 million people and 702,704 births. Of the births, three strata were defined: full term births (≥ 37 weeks’ gestation); pre-term births defined as before 37 weeks’ gestation; and high-risk pre-term births defined as before 37 weeks’ gestation and birth weight < 1,500 g. During the time period considered there were an average 641,510 and 61,194 births at ≥ 37 and < 37 weeks’ gestation, respectively, and an average 53,238 and 7,956 infants born pre-term with a birth weight ≥ 1500 g and < 1500 g, respectively [17–21].

### Vaccination schemes

For the current analysis, we focused on the National Immunization Program primary series and first booster for infants older than 12 months. In the current vaccination scheme, full-term and pre-term infants receive 4 doses of pentavalent vaccine (2, 4, 6 and 15 months of age), and 4 doses of IPV (2, 4 and 6 months and 5 years of age) [9]. We assumed that 50% of high-risk preterm infants receive 3 doses of the hexavalent vaccine (2, 4, and 6 months), 1 dose of pentavalent vaccine (15 months), and 1 dose of IPV (school entry), with the other 50% receiving 4 doses of pentavalent vaccine plus IPV (i.e. the same schedule as the other infants). The private insurance sector already vaccinates all eligible infants with the hexavalent vaccine for all primary series and first booster (2, 4, 6 and 15 months of age) regardless of whether they were born full-term or pre-term, including those considered high-risk (consistent with the alternative vaccination scheme). In the alternative vaccination scheme, the hexavalent vaccine was used for all primary series and first booster (at 2, 4, 6 and 15 months of age) for all eligible infants. Vaccination coverage was taken from 2015 to 2019 (Supplementary Table S1) [22].

The infant population was segmented according to coverage (where the vaccine was received in the Argentinean three-tier health system [23]: public, social security and private insurance sectors (Table 1)). The social security sector includes provincial, national union & other health maintenance organizations, and PAMI for elderly & retirees (the latter not included in the current analysis). To obtain a weighting for healthcare costs, the percentage of infants in the different sub-systems was considered to be: 37% public sector, 57% social security sector, 6% private insurance sector [24]. In our analysis, the latter two sectors (social security and private insurance) are combined and referred to as the private insurance/pre-paid sector henceforth. The number of doses distributed by the Ministry of Health corresponds to the total infant population eligible for vaccination (all newborns registered for one year). The total doses distributed by the Ministry of Health was adjusted by coverage rate but did not include the number of doses distributed in the private insurance sector.

**Table 1:** Vaccine distribution with the two schemes across the public and private sectors

Scheme	1st dose 2 months		2nd dose 4 months		3rd dose 6 months			Booster		
	Percentage	n	Percentage	n	Percentage	n		Percentage	n	
Public sector^†^	94%	660,542	94%	660,542	94%		660,542	94%		660,542
Private sector^†^	6%	42,162	6%	42,162	6%		42,162	6%		42,162
Public sector-vaccination coverage	90%	594,488	89%	587,882	88%		581,277	89%		587,882
1. Full-term > 37 wks	91.29%	542,717	91.29%	536,687	91.29%		530,657	91.29%		536,687
2. Pre-term < 37 wks > 1500 g	7.58%	45,039	7.58%	44,539	7.58%		44,308	7.58%		44,539
3. ^#^Pre-term < 1500 g (pentavalent plus IPV)	0.57%	3,365	0.57%	3,328	0.57%		3,291	1.13%		6,656
4. ^#^Pre-term < 1500 g (all hexavalent)	0.57%	3,365	0.57%	3,328	0.57%		3,291			
Private sector- vaccination coverage	100%	42,162	100%	42,162	100%		42,162	100%		42,162
5. Full-term > 37 wks	91.29%	38,491	91.29%	38,491	91.29%		38,491	91.29%		38,491
6. Pre-term < 37 wks > 1500 g	7.58%	3,194	7.58%	3,194	7.58%		3,194	7.58%		3,194
7. Pre-term < 1500 g	1.13%	477	1.13%	477	1.13%		477	1.13%		477

Entire cohort (n = 702,704) G, grams; wks, weeks

^†^The ministry of health in Argentina acquires and distributes vaccines for the National Immunization Program for 94% of the covered population regardless of their healthcare affiliation (i.e. both the public and social security sectors), the administration of which is free, with the exception of those in the private insurance sector who account for rest (6%)

^#^Coverage disaggregated for the current scheme. Both groups 3 and 4 shown in the table all received hexavalent vaccine in the alternative scheme

It was assumed that the effectiveness of the current and alternative vaccination schemes was the same, and this effectiveness was maintained with periodic boosters [25–30].

### Adverse events

The adverse event rate following vaccination with vaccines containing wP and aP included in the analysis were taken from Decker et al. (Supplementary Table S2) [31], and extrapolated to the vaccination cohorts assessed. However, since the Decker et al. study did not analyze the rate of adverse events following the booster, we arbitrarily assumed that this would be the same as that after the third dose of DTP. Although adverse event rates with vaccines containing wP and aP from Zhang et al., [32] and Patterson et al., [33] were considered, we chose to use those by Decker et al. [31] to be conservative since the latter generally reported a smaller reduction in the adverse event rates with aP-containing compared to wP-containing vaccines (i.e. a smaller difference in adverse event reporting rates). For seizures and other neurological effects such as hypotonic-hyporesponsiveness, data were taken from ACIP [34] and Cody et al. [35]: the rate of seizure (with or without fever) and hypotonia-hyporesponsiveness episodes with wP containing vaccines was estimated to be 1 case/1,750 vaccinated (i.e. 0.57 cases /1,000 doses) and that with aP containing vaccines to be 0.12/1,000 doses [34, 35].

### Parameters and associated costs

The parameters and associated costs included in our analysis are summarized in Table 2. It was assumed that some of the associated adverse events would give rise to health resource use (visits to the outpatient clinic, subsequent follow-up visits, emergency room visits and hospitalizations) and parental resources or actions (travel to clinic, work absenteeism). The cost impact derived from adverse event management was estimated by multiplying the proportion of affected infants for each adverse event (Table S2; according to Decker et al. [31]) with the rates obtained from the Delphi survey of 30 pediatricians (involved with inpatient or outpatient care) in Uruguay where they expressed their attitudes as well as those of the parents related to management of each respective adverse event (i.e. percent requiring visits to the outpatient clinic, the emergency room and hospitalization, follow-up visits, use of medication, parental resources/actions) according to gestational age and vaccine dose administered (Tables S3–S6). These estimates, as provided by the pediatricians, were dependent on whether the infant child was eutrophic or preterm with < 37 weeks’ gestation. For length of stay if hospitalized, a conservative approach of 1 day of hospitalization was estimated in all cases. Health system unit costs (differentiated for public, social security and private sectors) related to resource utilization due to adverse events associated with outpatient clinic visits, subsequent follow-up visits, emergency room visits, and hospitalizations were obtained from an acquired unit costs and health events IQVIA database (Table 2). In each case, parental costs related to travel to clinic and absenteeism were considered, as well as for medication usually recommended at home or in an outpatient clinic (analgesics/antipyretics [ibuprofen], and antiemetics [metochlopramide]). For costs associated with parental work absenteeism (Table 2), it was assumed that a family member lost half a working day in the outpatient clinic and emergency room, and one and half days for hospitalization, considering an employment rate of 41.5% [36]. Programmatic costs considered included labor, the supply chain, service delivery and capital costs from Portnoy et al. [37].

**Table 2:** Parameters and associated unit costs included in the analysis

Variables	Arg Pesos	US$	Source
**Analgesics/antipyretics use**			
Ibuprofen suspension: 2% suspension 2 g/100ml (Pfizer)	307.87	3.03	Kairos. Price update 10/04/2021 [47]
Ibuprofen suspension: FEBRATIC Ped. 2% (Roemmers)	294.49	2.89	Kairos. Price update: 09/30/2021 [48]
Ibuprofen suspension. PEDIATRIC ACTRON 2% Oral (Bayer)	302.00	2.97	Kairos. Price update: 10/12/2021 [49]
Ibuprofen suspension: Weight average*	301.45	2.96	
**Antiemetics use**			
Metochlopramide: RELIVERAN for CHILDREN 2 mg/ml. Oral drops x20ml (Gador)	544.51	5.35	Kairos. Price update: 10/18/2021 [50]
Metoclopramide: Vannier 5 mg /ml. Oral Drops x 20ml. (Vannier)	561.55	5.52	Kairos. Price update: 10/14/2021 [51]
Metochlopramide: Gastrocalm 2 mg/ml. Oral Drops x 20ml. (Cassará)	492.57	4.84	Kairos. Price update: 10/11/2021 [52]
Metochlopramide: Oral antiemetic drops x 20ml (weighted average)*	532.88	5.24	
**Weighted pediatric outpatient visit**			IQVIA Argentina. Unit Costs and Health Events Base [53]
Pediatric outpatient visit average: public sector (37% of system users)	609	5.99	[53]
Pediatric outpatient visit average: social security as a whole (57% of system users)	904	8.88	[53]
Pediatric outpatient visit average: private sector (6% of system users)	1.227	12.06	[53]
Weighted pediatric outpatient visit (100% of system users)	814	8.00	[53]
**Visit to pediatric emergency**			IQVIA Argentina. Unit Costs and Health Events Base [53]
Visit to pediatric emergency average: public sector (37% users of the system)	1.398	13.74	[53]
Pediatric emergency visit average: social security as a whole (57% of system users)	1.358	13.35	[53]
Visit to pediatric emergency average: private sector (6% users of the system)	1.914	18.81	[53]
Visit to pediatric emergency Weighted average (100% of system users)*	1.406	13.82	[53]
**Day of hospitalization in pediatric ward**			IQVIA Argentina. Unit Costs and Health Events Base [53]
Day of hospitalization in pediatric ward average: public sector (37% users of the system)	12,523	123.08	
Day of hospitalization in pediatric ward average: social security as a whole (57% users of the system)	18,649	183.28	[53]
Day of hospitalization in pediatric ward average: private sector (6% users of the system)	54,278	533.44	[53]
Day of hospitalization in pediatric ward weighted average (100% users of the system)	18,520	182.02	[53]
**Parental costs**			
Average cost of 1 bus transfer	14.3	0.14	Fare and social fare up to 12 km [54]
Cost of 1 working day (absenteeism) Average monthly income 26,021 per 24 daily wages	450	4.42	Employment rate 41.5% [36]
Cost of ½ working day (absenteeism)	225	2.21	Employment rate 41.5% [36]
**Cost of vaccines**			
Price of Pentavalent Vaccine Revolving Fund 2021. Bottle 1 liquid dose		1.0276	OPS Revolving Fund [55]
Price of IPV Revolving Fund 2021. Bottle 5 doses		3.1	OPS Revolving Fund [55]
Price of Hexavalent Revolving Fund 2021. Bottle 1 dose		21.12	OPS Revolving Fund [55]
Cost of Hexavalent vaccine private healthcare sector Hexaxim (Sanofi). Discount rate 40%	8,817.92	86.66	Kairos. Update date 09/30/2021 [56]
Cost of Hexavalent vaccine private healthcare sector Infanrix Hexa (GSK). Discount rate 40%	8,510.27	83.64	Kairos. Update date 10/14/2021 [57]
Cost of Hexavalent vaccine private health sector average. Discount rate 40%	8,664.09	85.15	
Programmatic costs per dose applied. Harvard Analysis (B&MGF)		3.18	Portnoy A et al. [37]

*weighted average is calculated by the mean

Prices were converted from Argentinean Pesos into US$ using an exchange rate of Arg Pesos 101.75 = US$1 (October 26, 2021) [38]

All costs were in 2021 prices. Vaccines and programmatic costs were listed in US dollars (US$); all other costs are listed in Argentinean pesos ($ Ars) and converted to US$ according to the exchange rate set (Arg Pesos 101.75 = US$1) by Banco Nación (average purchase-sale) on October 26th, 2021 [38] (Table 2).

A sensitivity analysis was carried out based on two vaccine coverage rate extremes: one where vaccination coverage was impacted by the COVID-19 pandemic (minimum coverage as occurred in 2020 due to the pandemic [average DTP1–3 coverage, 77% and for the fourth dose, 66%]) [39]; and the other where the recommended target coverage (95%) for each dose was assumed.

## Results

### Base scenario cost analysis

**Fig. 1:**
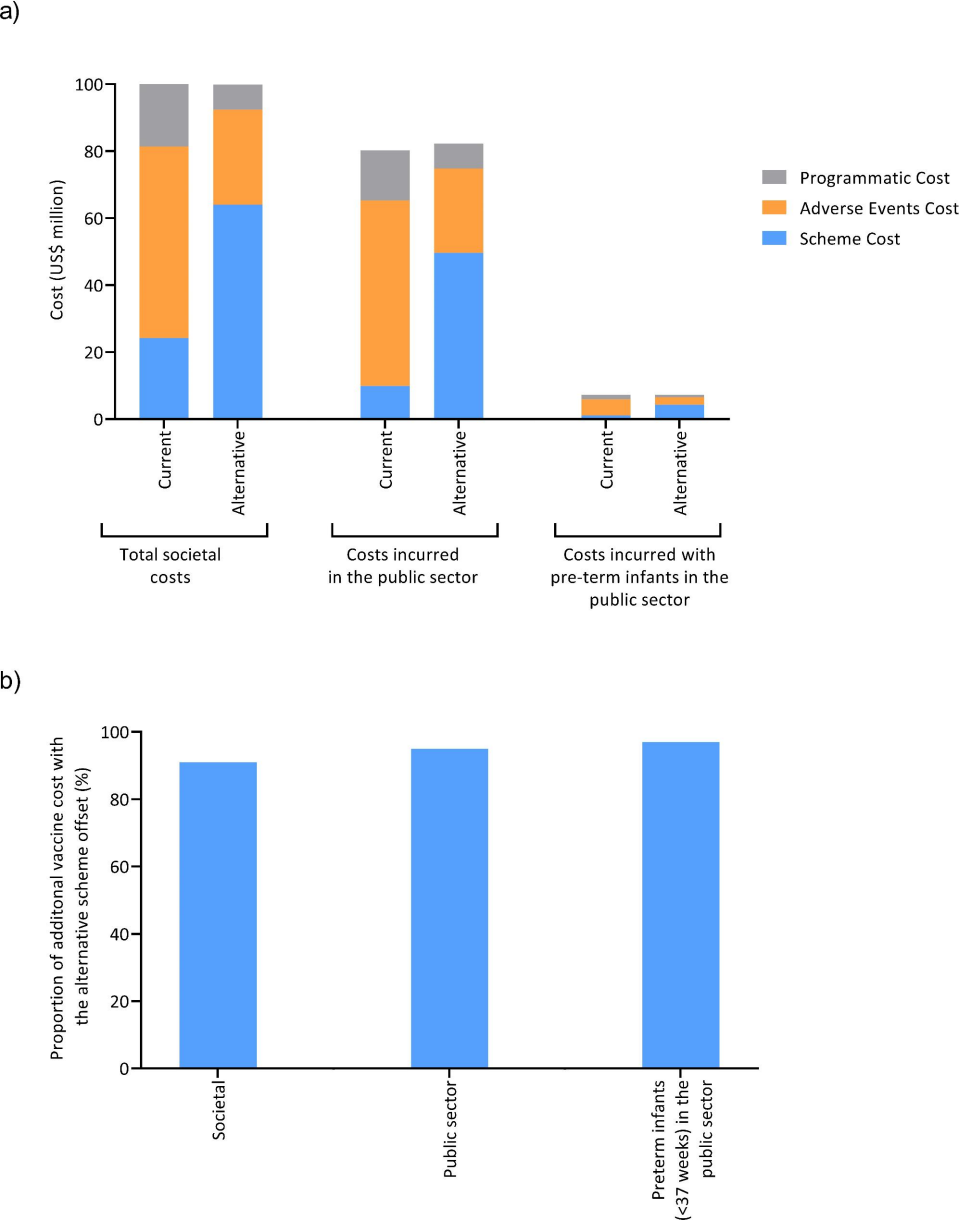
Summary of the costs of the current and alternative schemes (**a**), and the proportion of additional vaccine costs associated with the alternative scheme offset by fewer AE-associated costs and lower programmatic costs (**b**) Current scheme (pentavalent; DTwP-Hib-HB plus IPV) and alternative (hexavalent; DTaP-Hib-HB-IPV)

The costs of the current and alternative vaccination schemes from the societal perspective are summarized in Table 3. Although the cost of vaccines with the alternative scheme would be US$39.8 million (M) more than with the current scheme, these additional costs are in large part offset by fewer adverse event-associated costs (US$ − 28.8 M) and lower programmatic costs (US$ − 7.4 M) such that the overall cost of the alternative scheme would only be an additional US$3.6 M from the societal perspective (Table 3/Fig. 1).

**Table 3:** Summary of the costs for the current and alternative schemes from the societal perspective

Summary of adverse event cost	Total cost for AE category	Total cost per vaccine category	Programmatic total	Total
**Current scheme (pentavalent vaccine + IPV**)				
Cost for 1st dose at 2 months	18,444,071	6,101,143	3,770,239	28,315,453
Cost for 2nd dose at 4 months	14,811,174	6,073,243	3,728,347	24,612,764
Cost for 3rd dose at 6 months	12,266,659	6,045,343	3,686,456	21,998,458
Cost for booster dose (15–18 months)^**#**^	11,609,648	6,016,691	3,738,931	21,365,269
Scheme total	57,131,553	24,236,418	14,923,972	96,291,944
**Alternative scheme (hexavalent vaccine)**				
Cost for 1st dose at 2 months	8,139,643	16,145,726	1,890,471	26,175,840
Cost for 2nd dose at 4 months	5,893,507	16,006,219	1,869,465	23,769,192
Cost for 3rd dose at 6 months	6,482,325	15,866,713	1,848,460	24,197,498
Cost for booster dose (15–18 months)	7,847,865	16,006,219	1,876,024	25,730,108
Scheme total	28,363,340	64,024,878	7,484,420	99,872,638
Difference in total cost between schemes (alternative – current)	–28,768,213	39,788,460	–7,439,552	3,580,694

^#^Booster dose, for the purposes of costs calculations, the IPV was included in the vaccination scheme of each child at the same time as the 4th pentavalent dose at age 15–18 month

All costs are in US$; AE, adverse event

The comparative costs of the two vaccination schemes incurred through those in the public sector are summarized in Table 4. Similarly, although the cost of vaccines with the alternative scheme would be US$ 39.8 M more than with the current scheme, these additional costs are in large part offset by fewer adverse event-associated costs (US$ − 30.2 M) and lower programmatic costs (US$ − 7.4 M) such that the overall cost of the alternative scheme would only be an additional US$ 2.1 M (Table 4; Fig. 1) in the public sector.

**Table 4:** Summary of costs of the current and alternative schemes in the public sector

Summary of adverse event cost	Total cost for AE category	Total cost per vaccine category	Programmatic total	Total
**Current scheme (pentavalent vaccine + IPV)**				
Cost for 1st dose at 2 months	18,006,177	2,510,994	3,770,239	24,287,410
Cost for 2nd dose at 4 months	13,389,732	2,483,094	3,728,347	20,601,174
Cost for 3rd dose at 6 months	11,826,718	2,455,195	3,686,456	17,968,368
Cost for booster dose (15–18 months)^**#**^	11,199,664	2,426,542	3,738,931	17,365,137
Scheme total	55,422,291	9,875,826	14,923,972	80,222,090
**Alternative scheme (hexavalent vaccine)**				
Cost for 1st dose at 2 months	6,174,079	12,555,578	1,890,471	20,620,127
Cost for 2nd dose at 4 months	5,499,117	12,416,071	1,869,465	19,784,653
Cost for 3rd dose at 6 months	6,065,325	12,276,565	1,848,460	20,190,350
Cost for booster dose (15–18 months)	7,437,880	12,416,071	1,869,465	21,723,417
Scheme total	25,176,401	49,664,285	7,477,861	82,318,548
Difference in total cost between schemes (alternative – current)	–30,245,890	39,788,459	–7,446,111	2,096,458

^**#**^Booster dose, for the purposes of costs calculations, the IPV was included in the vaccination scheme of each child at the same time as the 4th pentavalent dose at age 15–18 months

All costs are in US$; AE, adverse event

The comparative costs of the two vaccination schemes incurred through those in the public sector with pre-term infants (< 37 weeks’ gestation) are summarized in Table 5. Although the cost of vaccines with the alternative scheme would be US$2.7 M more than with the current scheme, these additional costs are in large part offset by fewer adverse event-associated costs (US$ − 2.6 M) and lower programmatic costs (US$ − 0.6 M) such that the overall cost of the alternative scheme would only be an additional US$84,023 (Table 5; Fig. 1) in the public sector.

**Table 5:** Summary of the costs and current and alternative schemes for preterm infants (< 37 weeks’ gestation) in the public sector

Summary of adverse event cost	Total cost for AE category	Total cost per vaccine category	Programmatic total	Total
**Current scheme (pentavalent vaccine + IPV)**				
Cost for 1st dose at 2 months	1,542,529	270,874	318,556	2,131,959
Cost for 2nd dose at 4 months	1,296,065	267,864	315,016	1,878,946
Cost for 3rd dose at 6 months	1,013,153	264,854	311,477	1,589,484
Cost for booster dose (pentavalent 15–18 months)^**#**^	982,841	211,312	325,600	1,519,753
Scheme total	4,834,588	1,014,905	1,270,649	7,120,142
**Alternative scheme (hexavalent vaccine)**				
Cost for1st dose at 2 months	551,588	1,093,385	164,629	1,899,602
Cost for 2nd dose at 4 months	486,879	1,081,237	162,800	1,730,916
Cost for 3rd dose at 6 months	536,618	1,069,088	160,971	1,766,677
Cost for booster dose (15–18 months)	652,934	1,081,237	162,800	1,896,971
Scheme total	2,228,020	4,324,946	651,199	7,204,165
Difference in total between schemes (alternative – current)	–2,606,568	3,310,041	–619,450	84,023

^**#**^Booster dose, for the purposes of costs calculations, the IPV was included in the vaccination scheme of each child at the same time as the 4th pentavalent dose at age 15–18 months

All costs are in US$; AE, adverse event

### Alternative scenario cost analysis

Alternative scenarios were considered to reflect two (lower and upper) plausible extremes in vaccination coverage; low coverage affected by the COVID-19 pandemic and the recommended target coverage (95%) for each dose were assumed (Table 6). In the two scenarios, the cost of vaccines with the alternative scheme ranged from US$33.1 M to US$42.5 M more than the current scheme, and these additional costs would also in large part be offset by fewer adverse event-associated costs and lower programmatic costs such that the overall cost of the alternative scheme would only be an additional US$2.5 to US$3.9 M from the societal perspective, with similar outcomes observed in the public sector.

**Table 6:** Alternate scenario analysis

Outcome indicators by vaccination coverage	Base-case analysis Average 2015–2019		Lower limit (COVID-19 pandemic affected vaccine coverage)		Upper limit a (95% vaccine coverage assumed)	
Health system	All sectors	Public sector	All sectors	Public sector	All sectors	Public sector
Total cost of current scheme	96.3	80.2	83.2	67.1	101.6	85.6
Total cost of alternative scheme	99.9	82.3	85.7	68.3	105.5	87.9
Increase in absolute value of total cost with the alternative scheme	3.6	2.1	2.5	1.2	3.9	2.3
Increase in percentage	3.7%	2.6%	3.0%	1.8%	3.8%	2.7%
Cost of vaccines in current scheme	24.2	9.9	22.6	8.2	24.9	10.6
Cost of vaccines in alternative scheme	64.0	49.7	55.7	41.3	67.4	53.0
Cost of adverse events in current scheme	57.1	55.4	48.2	46.5	60.8	59.1
Cost of adverse events in alternative scheme	28.4	25.5	23.8	20.8	30.1	26.9
Programmatic costs in current scheme	14.9	14.9	12.4	12.4	15.9	15.9
Programmatic cost in alternative scheme	7.5	7.5	6.2	6.2	8.0	8.0
Cost difference between vaccines	39.8	39.8	33.1	33.1	42.5	42.5
Difference reduction rate (minimization = increase in absolute value / difference cost of the current scheme)	91.0%	94.7%	92.5%	96.4%	90.9%	94.6%
Cost of the current scheme per child	137.0	114.2	118.4	95.5	114.6	121.8
Cost of the alternative scheme per child	142.1	117.1	121.9	97.2	150.1	125.0
Increase in cost per child	5.1	3.0	3.5	1.7	5.5	3.3
Increase in % total cost per child	3.7%	2.6%	3.0%	1.8%	3.8%	2.7%
Cost of current vaccination schedule per child (4 doses per vaccine)	34.5	14.1	32.1	11.7	35.5	15.0
Cost of vaccines in alternative scheme per child (4 doses per vaccine)	91.1	70.7	79.2	58.8	95.9	75.4

All costs are in US$ million

## Discussion

In this study, we considered three infant populations in Argentina from the societal perspective: all infants up to 1 year of age (inclusive of the public, social security, and private sectors); and two sub-cohorts who currently receive vaccines in the public sector (distributed by the Ministry of Health); and specifically, preterm infants < 37 weeks’ gestation (including high-risk infants) in the public sector. We compared, across these infant populations, the costs associated with the current pertussis vaccination scheme with the pentavalent vaccine plus IPV (assuming that half of the high-risk infants received the pentavalent-based scheme and the other half the hexavalent scheme) with that of a hexavalent scheme covering the same diseases. We demonstrated, assuming both vaccination schemes had similar effectiveness, that switching from the current pentavalent vaccine plus IPV to the alternative scheme with the hexavalent vaccine, would result in higher vaccine costs in all populations assessed. The higher vaccine costs associated with the hexavalent vaccine scheme are mostly offset by reduced costs for adverse events associated with aP compared with wP in the current pentavalent vaccine, as well as improved logistics and programmatic costs associated with the reduction of vaccinations in the childhood series.

Previous studies found that a switch from the pentavalent vaccine plus IPV/oral polio vaccine to the hexavalent vaccine from the Chilean and Argentinean societal perspectives would result in incremental costs of US$6.45 M and US$19.7 M, respectively [14, 40], which are much higher than in our current study. The study undertaken in Chile (and the previous Argentinean study) did not have programmatic costs from Portnoy et al. 2020 [37] available at the time of their analyses, but which we included. In addition, we also included preterm infants. Of note, a Delphi survey of pediatric experts from Uruguay was also available which assessed attitudes towards the management of each adverse event, including prescription drug and diagnostic test requirements, and rates of hospitalization following each dose of the vaccines (Unpublished data [in review]). The Delphi survey enabled us to improve the assessment of the ‘health system’s behavior’ in terms of medical care and use of resources when adverse events occur. These adjustments/refinements, to incorporate more up to date information may, in part, explain the larger differences observed in previous analyses compared with our study.

We did not take into consideration the risk of non-completion of the vaccination series due to parental apprehension, which is significantly higher in those who receive vaccines containing wP than those with aP [33]. Lower vaccine coverage could result in higher disease rates, especially for those diseases that need high vaccine coverage for prevention such as pertussis and *Haemophilus influenza* type B. The additional cases as a result of lower vaccine coverage and the associated costs were not considered. Additionally, the costs associated with delayed vaccinations, such as additional clinic visits, were not considered. Programmatic errors were also not considered in the current scheme and are more likely to occur when multiple vaccines are required [41, 42]. The current Argentinean vaccination schedule includes three vaccinations on the same day at the 2 and 4 month visits (pentavalent, IPV and pneumococcal conjugate 13 serotypes vaccine). The simultaneous administration of several vaccines can lead to physicians omitting one vaccine due to lack of the product, family reluctance and child discomfort; missed vaccinations due to these reasons were not included in the analysis [43, 44]. Additionally, parental absenteeism and family transfer to the healthcare center were considered for only one parent; travel with additional family members could result in added costs. Costs associated with informal workers, who do not register income or contributions to social security, were also not considered. As such, our analysis may have underestimated the benefits of switching to DTaP-Hib-HB-IPV.

We assumed the effectiveness of both wP and aP vaccines was the same. Previous studies suggest that waning immunity in children who received aP vaccines occurs more rapidly than those who received wP vaccines [45, 46]. However, it is not apparent how waning immunity affects pertussis incidence rates, particularly in children < 5 years of age. In addition, countries where only wP vaccines have been used have similar numbers of pertussis cases as countries using aP vaccines [29]. Additionally, it has been demonstrated that replacement of wP combination vaccines with aP combination vaccines enhances immunity until 6 years of age, when children receive preschool boosters [46]. As such, similar effectiveness was a reasonable assumption to prevent bias in the economic estimate.

The risk of minor adverse events such as fever, irritability, uncontrollable crying, vomiting, pain, hardening, redness and edema after vaccination are significantly lower in those who receive aP vaccines compared to wP vaccines [31–33]. Similarly, serious adverse events such as seizures, hyporesponse-hypotonia syndrome and apnea are less frequently reported with aP vaccines than with wP vaccines [31–33]. Although the incidence of serious adverse events is rare, they are a cause for parental concern and can contribute to vaccine hesitancy and loss of public trust in vaccines. As such, the more favorable safety profile of aP containing vaccines over those with wP would help alleviate parental concerns and reduce vaccine hesitancy [31–33].

Our base scenario assessed the 5 years before the COVID-19 pandemic (2015–2019). Similar marginally higher costs were estimated with the hexavalent vaccine scheme in sensitivity scenarios assessed, where vaccine coverage was assumed to be affected by the COVID-19 pandemic (low coverage) or when 95% coverage for each dose was assumed (recommended coverage).

In conclusion, a switch to the hexavalent vaccine scheme in Argentina would lead to marginal additional costs to society.

### Electronic supplementary material

Below is the link to the electronic supplementary material.

Supplementary Material 1

## Data Availability

All data generated or analysed during this study are included in this published article.
