# Supplementary material for: Cost minimization analysis of a hexavalent vaccine in Argentina
Source: BMC Health Serv Res. 2023 Oct 6;23:1067. doi: 10.1186/s12913-023-10038-0 (PMC10557326; doi:10.1186/s12913-023-10038-0)
Supplement: Supplementary file 1 — Supplementary Material 1 [file 12913_2023_10038_MOESM1_ESM.docx]

**Supplementary materials**

Table S1. Vaccine coverage considered in the current analysis [1]

| Vaccination coverage (%) | 2015 | 2016 | 2017 | 2018 | 2019 | Average |
| --- | --- | --- | --- | --- | --- | --- |
| DTP1 | 94 | 88 | 94 | 91 | 82 | 90 |
| DTP2^#^ | 94 | 90 | 91 | 88 | 80 | 89 |
| DTP3 | 94 | 92 | 88 | 86 | 78 | 88 |
| Polio3 | 93 | 87 | 85 | 84 | 78 | 85 |
| Booster* | 89 | 90 | 90 | 94 | 82 | 89 |

DTP1, first DTP dose; DTP2, second DTP dose; DTP3, third DTP dose; Polio3, third polio dose

^#^For DPT2, an average of DPT1 and DPT3 was calculated.

*For the DPT booster in the second year of life, Measles, Mumps & Rubella (MMR1) coverage was considered.

Table S2. Adverse events reported after administration of vaccines containing either whole-cell pertussis or acellular pertussis components [2]

| Adverse events (Decker et al) | 1^st^ dose | | 2^nd^ dose | | 3^rd^ dose | |
| --- | --- | --- | --- | --- | --- | --- |
|  | Whole cell | Acellular | Whole cell | Acellular | Whole cell | Acellular |
| Fever 100.1 to 101^o^F (37.8^o^C to 38.38^o^C) | 24.3% | 2.3% | 28.8% | 12.8% | 27.8% | 15.2% |
| Fever 101.1 to 102^o^F (38.39^o^C to 38.89^o^C) | 3.0% | 0.8% | 3.9% | 0.8% | 7.3% | 2.3% |
| Fever >102^o^F (38.89^o^C or higher) | 0.0% | 0.0% | 1.4% | 0.8% | 2.6% | 0.0% |
| Redness 1 to 20 mm | 40.8% | 13.5% | 41.6% | 24.1% | 44.4% | 25.8% |
| Redness >20 mm | 8.6% | 0.8% | 6.1% | 1.5% | 3.2% | 3.0% |
| Swelling 1 to 20 mm | 23.2% | 7.5% | 26.6% | 16.5% | 30.1% | 14.4% |
| Swelling >20 mm | 16.5% | 0.8% | 9.5% | 0.8% | 5.6% | 3.8% |
| Moderate pain | 17.6% | 2.3% | 12.6% | 3.0% | 12.0% | 3.8% |
| Severe pain | 9.7% | 0.0% | 6.1% | 0.0% | 3.8% | 0.0% |
| Moderate irritation | 16.8% | 3.8% | 16.5% | 6.0% | 12.6% | 4.5% |
| Severe irritation | 3.8% | 0.8% | 7.0% | 0.8% | 4.7% | 0.8% |
| Somnolence | 43.5% | 28.6% | 31.0% | 14.3% | 24.6% | 14.4% |
| Anorexia | 19.5% | 7.5% | 16.5% | 4.5% | 14.3% | 12.1% |
| Vomiting | 7.0% | 3.0% | 4.5% | 1.5% | 5.3% | 3.8% |

Table S3. Proportion with adverse events who would seek medical consultation by adverse event experienced and vaccination dose. Rates were obtained from a Delphi survey of 30 pediatricians (involved with inpatient or outpatient care) in Uruguay where they expressed their attitudes as well as those of the parents related to management of the respective adverse events

| Adverse events | 1^st^ dose | 2^nd^ dose | 3^rd^ dose | Booster |
| --- | --- | --- | --- | --- |
| Fever 100.1 to 101^o^F (37.8^o^C to 38.38^o^C) | 16% | 18% | 12% | 9% |
| Fever 101.1 to 102^o^F (38.39^o^C to 38.89^o^C) | 34% | 32% | 31% | 27% |
| Fever >102^o^F (38.89^o^C or higher) | 50% | 50% | 48% | 41% |
| Redness 1 to 20 mm | 23% | 16% | 13% | 11% |
| Redness >20 mm | 46% | 45% | 39% | 33% |
| Swelling 1 to 20 mm | 19% | 15% | 15% | 13% |
| Swelling >20 mm | 41% | 43% | 37% | 34% |
| Moderate pain | 24% | 23% | 22% | 19% |
| Severe pain | 68% | 62% | 63% | 64% |
| Severe irritation | 60% | 74% | 74% | 74% |
| Somnolence | 65% | 69% | 71% | 67% |
| Anorexia | 30% | 29% | 22% | 16% |
| Vomiting | 45% | 46% | 41% | 38% |
| Convulsions | 100% | 97% | 98% | 100% |
| Hypotonia-hyporesponsiveness | 98% | 93% | 94% | 94% |

Table S4. Proportion with adverse events who would be referred to the emergency room (including those who require consultation with an emergency room specialist and hospitalization) by adverse event experienced and vaccination dose. Rates were obtained from a Delphi survey of 30 pediatricians (involved with inpatient or outpatient care) in Uruguay where they expressed their attitudes as well as those of the parents related to management of the respective adverse events

| Adverse events | 1^st^ dose | 2^nd^ dose | 3^rd^ dose | Booster |
| --- | --- | --- | --- | --- |
| Redness 1 to 20 mm | 3% | 0% | 0% | 0% |
| Redness 1 to 20 mm (if premature, <37 WGA) | 3% | 0% | 0% | 0% |
| Redness >20 mm | 20% | 13% | 13% | 10% |
| Redness >20 mm (if premature, <37 WGA) | 20% | 17% | 13% | 13% |
| Swelling 1 to 20 mm | 7% | 3% | 3% | 3% |
| Swelling 1 to 20 mm (if premature, <37 WGA) | 7% | 3% | 3% | 3% |
| Swelling >20 mm | 27% | 13% | 20% | 13% |
| Swelling >20 mm (if premature, <37 WGA) | 27% | 13% | 20% | 13% |
| Swelling >20 mm (require ER specialist) | 0% | 0% | 3% | 0% |
| Swelling >20 mm (require ER specialist if premature, <37 WGA) | 0% | 0% | 3% | 0% |
| Swelling >20 mm (require hospitalization after ER specialist) | 3% | 0% | 0% | 0% |
| Moderate pain | 10% | 10% | 7% | 3% |
| Moderate pain (if premature, <37 WGA) | 10% | 10% | 7% | 3% |
| Severe pain | 67% | 33% | 57% | 47% |
| Severe pain (if premature, <37 WGA) | 70% | 37% | 60% | 53% |
| Persistent high-pitched cry or irritability | 57% | 40% | 47% | 40% |
| Persistent high-pitched cry or irritability (if premature, <37 WGA) | 60% | 47% | 50% | 40% |
| Persistent high-pitched cry or irritability (require ER specialist) | 3% | 0% | 0% | 0% |
| Persistent high-pitched cry or irritability (require ER specialist if premature, <37 WGA) | 3% | 0% | 0% | 0% |
| Severe irritation | 53% | 70% | 60% | 57% |
| Severe irritation (if premature, <37 WGA) | 57% | 70% | 60% | 57% |
| Severe irritation (require ER specialist) | 7% | 3% | 0% | 0% |
| Severe irritation (require ER specialist if premature, <37 WGA) | 7% | 3% | 0% | 0% |
| Somnolence | 73% | 63% | 60% | 63% |
| Somnolence (if premature, <37 WGA) | 77% | 67% | 60% | 63% |
| Somnolence (require ER specialist) | 3% | 0% | 0% | 0% |
| Somnolence (require ER specialist if premature, <37 WGA) | 3% | 0% | 0% | 0% |
| Anorexia | 20% | 10% | 7% | 3% |
| Anorexia (if premature, <37 WGA) | 27% | 13% | 10% | 3% |
| Vomiting | 50% | 50% | 43% | 37% |
| Vomiting (if premature, <37 WGA) | 50% | 53% | 47% | 40% |
| Convulsions | 93% | 93% | 93% | 90% |
| Convulsions (if premature, <37 WGA) | 93% | 93% | 93% | 90% |
| Convulsions (require ER specialist) | 17% | 0% | 0% | 0% |
| Convulsions (require ER specialist if premature, <37 WGA) | 17% | 0% | 0% | 0% |
| Convulsions (require hospitalization after ER specialist) | 40% | 40% | 37% | 37% |
| Hypotonia-hyporesponsiveness | 100% | 97% | 97% | 87% |
| Hypotonia-hyporesponsiveness (if premature, <37 WGA) | 100% | 97% | 97% | 87% |
| Hypotonia-hyporesponsiveness (require ER specialist) | 17% | 0% | 0% | 0% |
| Hypotonia-hyporesponsiveness (require ER specialist if premature, <37 WGA) | 17% | 0% | 0% | 0% |

ER, emergency room

WGA, weeks’ gestation age at birth

Table S5. Proportion with adverse events who would need follow-up by adverse event experienced and vaccination dose. Rates were obtained from a Delphi survey of 30 pediatricians (involved with inpatient or outpatient care) in Uruguay where they expressed their attitudes as well as those of the parents related to management of the respective adverse events

| Adverse events | 1^st^ dose | 2^nd^ dose | 3^rd^ dose | Booster |
| --- | --- | --- | --- | --- |
| Fever 100.1 to 101^o^F (37.8^o^C to 38.38^o^C) | 63% | 47% | 30% | 33% |
| Fever 100.1 to 101^o^F (37.8^o^C to 38.38^o^C) (if premature, <37 WGA) | 67% | 50% | 33% | 33% |
| Fever 101.1 to 102^o^F (38.39^o^C to 38.89^o^C) | 60% | 53% | 50% | 53% |
| Fever 101.1 to 102^o^F (38.39^o^C to 38.89^o^C) (if premature, <37 WGA) | 67% | 60% | 53% | 53% |
| Fever >102^o^F (38.89^o^C or higher) | 43% | 57% | 57% | 60% |
| Fever >102^o^F (38.89^o^C or higher) (if premature, <37 WGA) | 57% | 60% | 60% | 60% |
| Redness 1 to 20 mm | 47% | 53% | 53% | 50% |
| Redness 1 to 20 mm (if premature, <37 WGA) | 47% | 53% | 53% | 50% |
| Redness >20 mm | 67% | 70% | 53% | 50% |
| Redness >20 mm (if premature, <37 WGA) | 73% | 70% | 60% | 53% |
| Redness >20 mm (require specialist) | 3% | 0% | 0% | 0% |
| Redness >20 mm (require specialist if premature, <37 WGA) | 3% | 0% | 0% | 0% |
| Swelling 1 to 20 mm | 47% | 63% | 50% | 43% |
| Swelling 1 to 20 mm (if premature, <37 WGA) | 47% | 63% | 50% | 43% |
| Swelling >20 mm | 67% | 63% | 60% | 53% |
| Swelling >20 mm (if premature, <37 WGA) | 67% | 67% | 60% | 53% |
| Moderate pain | 43% | 47% | 43% | 40% |
| Moderate pain (if premature, <37 WGA) | 43% | 47% | 43% | 40% |
| Moderate pain (require specialist) | 3% | 0% | 0% | 3% |
| Moderate pain (require specialist if premature, <37 WGA) | 3% | 0% | 0% | 3% |
| Severe pain | 40% | 57% | 57% | 40% |
| Severe pain (if premature, <37 WGA) | 43% | 60% | 57% | 47% |
| Severe pain (require specialist) | 7% | 0% | 0% | 3% |
| Severe pain (require specialist if premature, <37 WGA) | 7% | 0% | 0% | 3% |
| Persistent high-pitched cry or irritability | 53% | 57% | 60% | 40% |
| Persistent high-pitched cry or irritability (if premature, <37 WGA) | 53% | 57% | 60% | 40% |
| Severe irritation | 47% | 33% | 47% | 47% |
| Severe irritation (if premature, <37 WGA) | 47% | 33% | 47% | 47% |
| Somnolence | 40% | 40% | 37% | 30% |
| Somnolence (if premature, <37 WGA) | 40% | 40% | 37% | 30% |
| Anorexia | 50% | 50% | 50% | 53% |
| Anorexia (if premature, <37 WGA) | 53% | 50% | 53% | 57% |
| Vomiting | 53% | 43% | 57% | 57% |
| Vomiting (if premature, <37 WGA) | 55% | 43% | 57% | 60% |
| Vomiting (require specialist) | 0% | 0% | 0% | 0% |
| Vomiting (require specialist if premature, <37 WGA) | 3% | 0% | 0% | 0% |
| Convulsions | 73% | 40% | 33% | 40% |
| Convulsions (if premature, <37 WGA) | 73% | 40% | 33% | 40% |
| Convulsions (require specialist) | 20% | 10% | 0% | 7% |
| Convulsions (require specialist if premature, <37 WGA) | 20% | 17% | 0% | 10% |
| Hypotonia-hyporesponsiveness | 53% | 43% | 37% | 33% |
| Hypotonia-hyporesponsiveness (if premature, <37 WGA) | 53% | 43% | 37% | 33% |
| Hypotonia-hyporesponsiveness (require specialist) | 7% | 20% | 0% | 7% |
| Hypotonia-hyporesponsiveness (require specialist if premature, <37 WGA) | 13% | 20% | 0% | 7% |

WGA, weeks’ gestation age at birth

Table S6. Proportion with adverse events who would require nonsteroidal anti-inflammatory drugs (NSAIDs)^#^ by adverse event experienced and vaccination dose. Rates were obtained from a Delphi survey of 30 pediatricians (involved with inpatient or outpatient care) in Uruguay where they expressed their attitudes as well as those of the parents related to management of the respective adverse events

| Adverse event | 1^st^ dose | 2^nd^ dose | 3^rd^ dose | Booster |
| --- | --- | --- | --- | --- |
| Fever 100.1 to 101^o^F (37.8^o^C to 38.38^o^C) | 100% | 100% | 100% | 93% |
| Fever 101.1 to 102^o^F (38.39^o^C to 38.89^o^C) | 97% | 100% | 100% | 97% |
| Fever >102^o^F (38.89^o^C or higher) | 100% | 97% | 100% | 100% |
| Redness 1 to 20 mm | 60% | 60% | 47% | 40% |
| Redness >20 mm | 60% | 60% | 60% | 63% |
| Swelling 1 to 20 mm | 47% | 43% | 37% | 40% |
| Swelling >20 mm | 40% | 50% | 37% | 50% |
| Moderate pain | 93% | 87% | 87% | 87% |
| Severe pain | 87% | 90% | 83% | 80% |
| Persistent high-pitched cry or irritability | 63% | 67% | 73% | 60% |
| Severe irritation | 73% | 57% | 53% | 53% |
| Somnolence | 7% | 10% | 7% | 13% |
| Anorexia | 3% | 13% | 20% | 13% |
| Vomiting | 7% | 7% | 3% | 10% |
| Convulsions | 10% | 20% | 27% | 33% |
| Hypotonia-hyporesponsiveness | 13% | 10% | 7% | 7% |

^#^Regarding other medication, antiemetics in the case of vomiting are indicated in 40%, 53%, 53% and 57%for each of the doses, respectively. In the case of seizures, benzodiazepines are indicated by 620%, 20%, 10% and 13% for each of the doses, respectively.

**References**

[1] Pan American Health Organization (PAHO). Immunization: Coverage data. Coverage reported in the Americas. Available at: [https://ais.paho.org/imm/IM_JRF_COVERAGE.asp (accessed 20 September 2022).

[2] Decker MD, Edwards KM, Steinhoff MC, Rennels MB, Pichichero ME, Englund JA, et al. Comparison of 13 acellular pertussis vaccines: adverse reactions. Pediatrics. 1995;96:557-66.
